# Poor sleep quality (PSQ) prevalence in patients with functional dyspepsia (FD): a systematic review and meta-analysis

**DOI:** 10.1097/MS9.0000000000003616

**Published:** 2025-07-25

**Authors:** Rashmikaa Netyam, Singam Shashank, Chiranjeevee R. Saravanan, Nikhil Deep Kolanu, Nanditha Karra, Sai Jahnu Sree Reddy Narla, Priyadarshi Prajjwal, Mohammed Dheyaa Marsool Marsool, Omniat Amir Hussin

**Affiliations:** a General Medicine, Hampshire Hospitals, NHS Foundation Trust, Basingstoke, UK; b Internal Medicine, Madras Medical College, India; c Internal Medicine, China Medical University, Shenyang, China; d Internal Medicine, Osmania Medical College, Hyderabad, India; e Internal Medicine, Mediciti Institute of Medical Sciences, India; f Neurology, Bharati Vidyapeeth University Medical College, Pune, India; g Internal Medicine, Mayo Clinic, Scottsdale, Phoenix, Arizona, USA; h Department of Medicine, Manhal University, Almanhal Academy for Science, Khartoum, Sudan

**Keywords:** functional dyspepsia, pooled prevalence, Rome criteria, sleep quality

## Abstract

**Background:**

Functional dyspepsia (FD), a disease of the gastroduodenal tract, is one of the functional gastrointestinal disorders (FGID) characterized by postprandial fullness and epigastric pain not attributed to any underlying organic diseases. Sleep quality refers to individuals’ satisfaction with their overall sleep, including sleep initiation, maintenance, duration, and feeling refreshed upon waking. Despite frequent associations between sleep disorders and FGID, comprehensive data on poor sleep quality (PSQ) in FD patients is lacking. Therefore, this study was conducted to investigate the occurrence of PSQ in patients with PSQ.

**Methods:**

PubMed, Scopus, and Web of Science were systematically searched to identify relevant literature. All studies reporting PSQ prevalence in FD patients or sufficient data to calculate it were included. Pooled prevalence was calculated for all studies, and an odds ratio was determined for studies with a healthy control group. Meta-regression and subgroup analyses assessed the moderating effects of different variables. Publication bias was examined using funnel plots and Egger’s test p-value. The work has been reported in line with AMSTAR (Assessing the methodological quality of systematic reviews) Guidelines.

**Results:**

To the best of our knowledge, this is the first study that pooled the estimate of prevalence of patients with FD with PSQ. Based on 2138 FD patients, the pooled prevalence of poor sleep quality is 57.2% (95% CI: 37%–75.2%), *I*^2^ = 98.4%. The asymmetric funnel plot and Egger’s test *P*-value indicated a significant publication bias for the pooled prevalence estimate. FD patients are at higher risk for poor sleep, with an odds ratio of 2.39 (95% CI: 1.86–3.06), *I*^2^ = 1.73%.

**Conclusion:**

Poor sleep is highly prevalent among FD patients, who are more susceptible to poor sleep than healthy individuals. The findings of the study should be interpreted with caution owing to the high heterogeneity and the publication bias observed for the pooled prevalence. Future research should standardize diagnostic parameters and investigate other confounding factors like anxiety and depression to achieve more accurate estimates.

## Introduction

Within the scope of functional gastrointestinal disorders (FGID), functional dyspepsia (FD) is a disease that still lacks a known etiology and is characterized by disruption of the normal functions of the gastroduodenal region due to an abnormal interaction between the brain and the gastrointestinal tract^[1,2]^. Functional dyspepsia is prevalent worldwide, affecting 9.80–20.20% of the general population from western countries and affecting 5.30–12.80% of the general population in eastern countries^[3]^. According to the Rome IV criteria for the diagnosis of different FGID, FD is diagnosed based on the symptoms of annoying or unpleasant postprandial fullness, annoying or unpleasant early sanitation, annoying or unpleasant burning in the epigastric region, or epigastric pain. In addition, the patient must not have evidence for any structural diseases that could cause this clinical picture and should have these manifestations for 6 months^[4]^. A bidirectional relationship has been found between functional dyspepsia and different psychological problems and disorders, most commonly depression and anxiety^[5,6]^. In addition, FD was observed to be linked with sleep disturbances, sleep disorders, and poor sleep quality^[7–9]^.

**HIGHLIGHTS**

- This study is the first to provide a pooled estimate of poor sleep quality (PSQ) prevalence in functional dyspepsia (FD) patients. The pooled prevalence of poor sleep quality in functional dyspepsia patients is 57.2%, based on data from 2138 patients across seven studies.
- FD patients are 2.39 times more likely to experience poor sleep than healthy individuals.
- Poor sleep may exacerbate FD symptoms, creating a vicious cycle that worsens both conditions.
- Differences in diagnostic criteria, data collection methods, and PSQI cut-offs led to variability in prevalence estimates.
- Future research should use objective sleep assessments, control for psychological factors, and standardize diagnostic criteria to improve accuracy.

Sleep quality could be defined in terms of the degree to which each individual is satisfied with the overall sleeping experience during the night, including early sleep initiation, sleep maintenance, total sleep duration, and a sense of refreshment after waking up^[10]^. Sleep quality could be subjectively assessed using different tools including the Pittsburgh sleep quality index, shortly known as the PSQI, which is a self-rated survey that examines different sleep dimensions during the last month, including subjective quality of sleep, early sleep initiation, sleep length, sleep disturbances, sleep efficiency daytime dysfunction, and usage of sleep medications^[11]^. In addition, a global sleep score could be used to divide the population into good sleep or poor sleep quality (PSQ) groups using a suitable cut-off value; hence, the prevalence of PSQ in different populations can be calculated.

**Figure 1.**
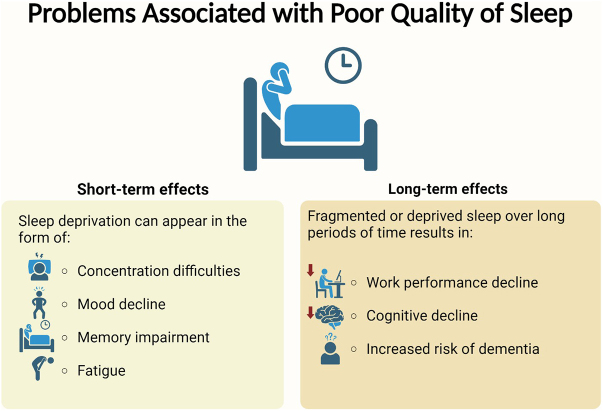
Short- and long-term outcomes of poor sleep quality and sleep deprivation.

The problem within PSQ is that it is linked with a poor quality of life, anxiety, and depression (Fig. 1)^[12,13]^. Moreover, PSQ has been shown to be one of the factors that disrupts GI motility, circadian rhythm, and normal stress response mechanisms, all of which are related to an increased susceptibility for functional gastrointestinal disorders and exacerbation of their symptoms^[14,15]^.

Therefore, we conducted this systematic review and meta-analysis with the aim of exploring the problem of PSQ in FD patients by estimating its pooled estimate of prevalence and comparing its occurrence between FD patients and healthy individuals using the odds ratio. This could possibly direct attention toward the quality of sleep as an important factor that should be considered when managing FD patients, so more effective and holistic treatment strategies could be developed.

## Methods

### Identification of eligible studies

The study design of this work is a meta-analysis for studies of prevalence. This work was done in order to examine the prevalence of PSQ in FD patients. We searched three databases systematically including PubMed, Web of Science (WOS), and Scopus. To collect relevant studies, a comprehensive search strategy was formed using a combination of words related to functional dyspepsia (functional dyspepsia, non-ulcer dyspepsia, indigestion, nervous dyspepsia, irritable stomach syndrome, pseudo-ulcer syndrome), and PSQ (poor sleep, Pittsburgh Sleep Quality Index, sleep quality, PSQI). All studies up to the cut-off date for conducting this search strategy, 25 August 2024, were included. Studies identified from each database were combined, and duplicated studies were identified and removed using Endnote software. The eligible studies were added to an Excel spreadsheet and subjected to screening.

### Development of the inclusion criteria and exclusion criteria

To be part of this analysis, identified studies should be: (1) conducted on humans, (2) observational in design (cohort, case–control, or cross-sectional studies), (3) reporting the prevalence of PSQ in FD patients or providing sufficient data to calculate the prevalence, (4) used subjective and validated methods for the calculation of PSQ prevalence, and (5) a full-text version of the study is available. Studies were excluded if they: (1) had an inappropriate study design, such as reviews, editorials, clinical trials, and book chapters, (2) were not conducted on patients with FD, (3) did not report the prevalence of PSQ in FD patients, and (4) were not in English language. Screening was performed in accordance with the 2020 PRISMA flow diagram for eligibility evaluation^[16]^.

### Extraction of data and methods of quality assessment

After identifying the eligible studies, the following items were identified and extracted from each one of the included studies: Family name (last name) of the first author, the year at which the study was published, country of study conduction, method of data collection, method of FD diagnosis, method of poor sleep assessment, mean age of the study participants, female percentage of the total study participants, total sample size, and the number of FD patients identified as having PSQ. If the study included a healthy control group, the total number of controls and those with PSQ were collected. Quality assessment was performed using an eight-item assessment instrument for single-arm cross-sectional studies, with a scale of 0–8. A summed overall score of 7 and 8 is regarded as high quality, a score range of 4–6 is regarded as an intermediate quality and the rest of the score is of low quality^[17]^. For studies having a healthy control group, the Newcastle-Ottawa scale (NOS) was used. It has eight questions, and the maximum overall score is 9. Studies having a summed score range of 7–9 are considered high quality. Scores 5 and 6 are considered intermediate quality. And the rest of the scale indicates poor quality^[18]^.

### Statistical analysis

The pooled prevalence of PSQ in FD patients with a 95% confidence interval was calculated based on all included studies, in addition to the calculation of the odds ratio for studies providing a healthy control group. *I*^2^ heterogeneity test was utilized to evaluate the between-study variance. *I*^2^ values greater than 75% are identified as severe heterogeneity and require the usage of the random-effect model. A random-effects model considers variations in study populations and collection methods^[19,20]^. As a result, a random-effects model was used for all statistical analyses. A sensitivity analysis was undertaken to determine whether one study inherently affected the overall estimate. An analysis based on different subgroups was performed to compare the pooled prevalence values in relation to the different study categorical groups. In this review, subgrouping was performed according to the method of FD diagnosis, PSQI cut-off value, and method of data collection. A meta regression analysis was done to assess the effect exerted by different quantitative variables on the reported prevalence. Both visual interpretation for funnel plot and the Egger’s test *P*-value assessment were used to investigate if there is an underlying publication bias. Duval and Tweedie’s trim and fill analysis were used in cases where a publication bias is statistically significant (Egger’s test *P*-value ≤ 0.05). In this study, Comprehensive Meta-Analysis software (3rd version) was the software used for performing all the statistics. A *P*-value less than or equal 0.05 was set as a threshold for statistical significance.

## Results

### Study identification and screening

**Figure 2.**
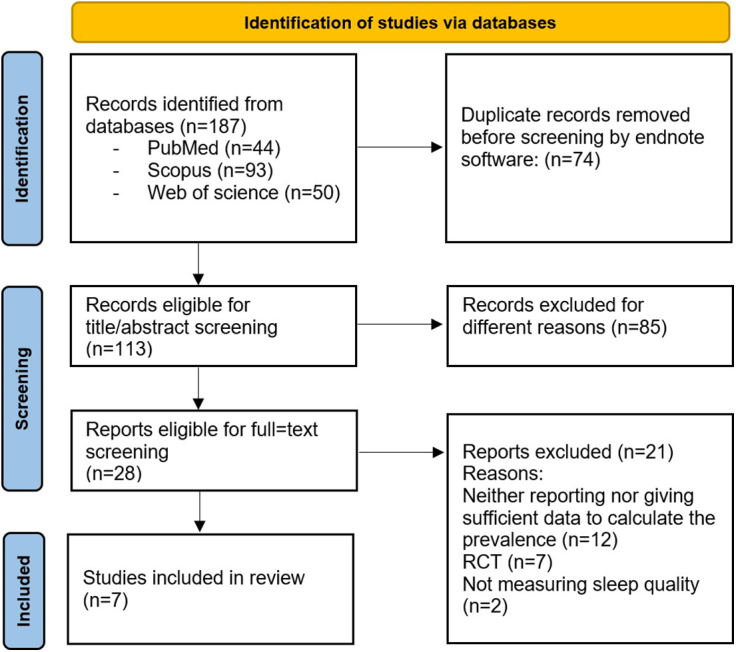
Graphical representation of the detailed results of database searching process.

Figure 2 shows the results of database searching and screening using the PRISMA flowchart. One hundred eighty-seven studies were identified by database searching, 74 of which were identified as duplicates and removed, leaving a total of 113 studies eligible for screening. Studies were excluded for not meeting the inclusion criteria previously mentioned in the methods, leaving only seven eligible studies to conduct this review on.

### Characteristics of studies included in this analysis

Table 1 shows the key characteristics of the seven studies within this review. These seven studies included a total of 2138 FD patients from four countries, including Japan, China, South Korea, and France. The smallest sample size among the seven studies was 101, while the largest was 920. The number of female patients was a subject of variety among the studies, ranging from 31.7% to 74.6%. FD was diagnosed by either Rome III or Rome IV criteria. PSQI was the only method utilized for the identification of PSQ in all seven studies with different cut-off values for dividing the population into good or poor sleep quality groups. Out of the seven included studies, only four studies included a healthy control group. The scores of the quality assessment for all the included studies indicate high or intermediate quality, full details about the results and scoring of different quality assessment items are shown in Table 2.

**Table 1:** Summarization of the characteristics of the included studies

Author and year	Country	Method of data collection	Function dyspepsia diagnosis	Sleep quality diagnosis	PSQI cut-off	Age (mean ± SD)	Female (%)	Total FD cases (*n*)	Poor sleep in FD (*n*)	Total control (*n*)	Poor sleep in control (*n*)	Study quality
Matsuzaki *et al.* 2018^[21]^	Japan	Interview	Rome III	PSQI	≥6	43.9 ± 10.1	46.5	202	101	257	88	8
Park *et al.* 2021^[22]^	South Korea	Questionnaire	Rome III	PSQI	≥8.5	55.4 ± 14.9	74.6	201	84	325	61	5
Shimpuku *et al.* 2014^[23]^	Japan	Questionnaire	Rome III	PSQI	>5.5	N/A	31.7	101	41	50	12	6
Wu *et al.* 2020^[24]^	China	Interview	Rome IV	PSQI	≥8	67.8 ± 8.4	35.6	214	103	N/A	N/A	6
Wuestenberghs *et al.* 2022^[25]^	France	Questionnaire	Rome IV	PSQI	>5	47 ± 15.7	76.6	355	289	N/A	N/A	6
Yamawaki *et al.* 2014^[26]^	Japan	Questionnaire	Rome III	PSQI	>5.5	N/A	68.9	145	51	44	8	7
Zhao *et al*. 2018^[27]^	China	Interview	Rome III	PSQI	>8	50.9 ± 11.5	62.07	920	801	N/A	N/A	5

**Table 2:** Quality assessment using the eight-item assessment instrument and the Newcastle-Ottawa Scale instrument

Eight-item assessment instrument for epidemiological studies									
First author, publication year	1. Target population is clearly defined?	2. Probability sampling OR entire population surveyed?	3. Is the response rate ≥80%?	4. Are non-responders clearly described?	5. Is the sample representative of the target population?	6. Were data collection methods standardized?	7. Were validated criteria used to diagnose poor sleep quality?	8. Are the prevalence estimates given with confidence intervals)?	Total score#
Wu *et al.* 2020	1	1	1	0	1	1	1	0	6 (Intermediate)
Wuestenberghs *et al.* 2022	1	1	0	1	1	1	1	0	6 (Intermediate)
Zhao *et al*. 2018	1	1	0	0	1	1	1	0	5 (Intermediate)
**Newcastle-Ottawa quality assessment scale**									
**First author, publication year**	**1. Is the case definition adequate**	**2. Representativeness of the cases**	**3. Selection of Controls**	**4. Definition of Controls**	**5. Comparability**	**6. Ascertainment of exposure**	**7. Same method of ascertainment for cases and controls**	**8. Non-Response rate**	Total score*
Matsuzaki *et al.* 2018	1	1	0	1	2	1	1	1	8 (High)
Park *et al.* 2021	1	1	0	1	1	0	1	0	5 (Intermediate)
Shimpuku *et al.* 2014	1	1	0	1	1	1	1	0	6 (Intermediate)
Yamawaki *et al.* 2014	1	1	1	1	1	1	1	0	7 (Intermediate)

### Pooled prevalence and odds ratio estimates

**Figure 3.**
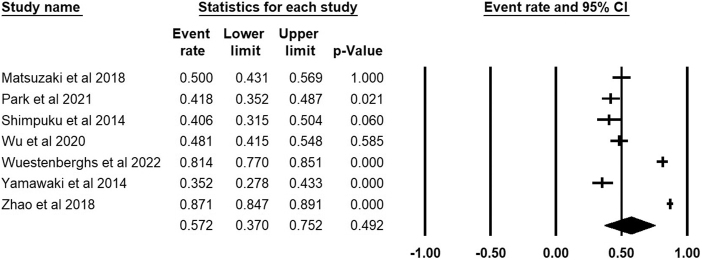
Forest plot of the prevalence of PSQ in FD patients with a 95% confidence interval.

**Figure 4.**
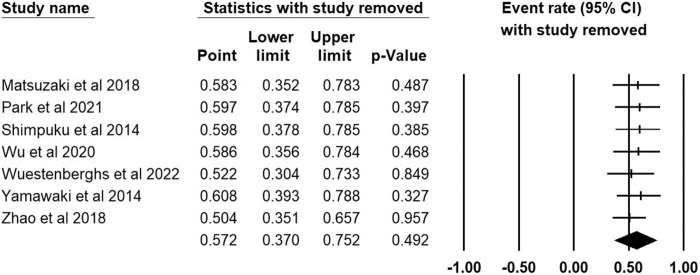
Sensitivity analysis of the pooled prevalence.

The forest plot of the prevalence of PSQ in FD patients, using a 95% confidence interval is shown in Figure 3. Based on 2138 FD patients from seven studies, the pooled prevalence of PSQ was 57.2% (95% CI: 37%–75.2%), *I*^2^ = 98.4%. Sensitivity analysis was conducted by sequentially removing one study and observing the changes in the pooled estimates, none of the included seven studies significantly affected the pooled prevalence estimates as shown in Figure 4.

**Figure 5.**
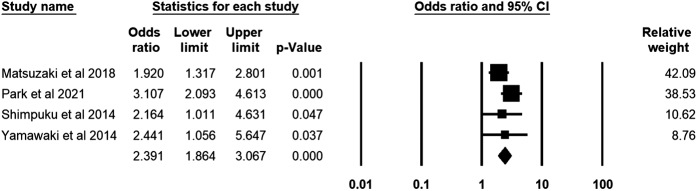
Forest plot of the odds ratio for PSQ in FD patients with a 95% confidence interval.

**Figure 6.**
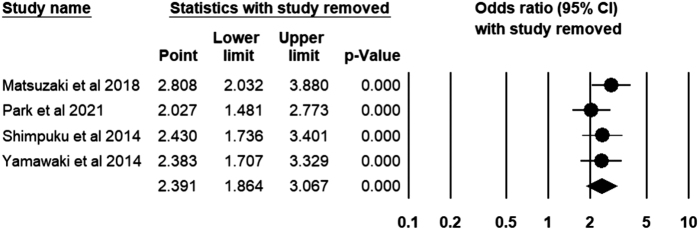
Sensitivity analysis of the pooled odds ratio.

For the four studies that included healthy controls, the odds ratio was calculated based on 649 patients with FD and 676 healthy controls. As shown in Figure 5, the calculated odds ratio was 2.39 (95% CI: 1.86–3.06), *I*^2^ = 1.73%. Figure 6 shows that none of the studies included significantly affected the pooled odds ratio estimates.

**Figure 7.**
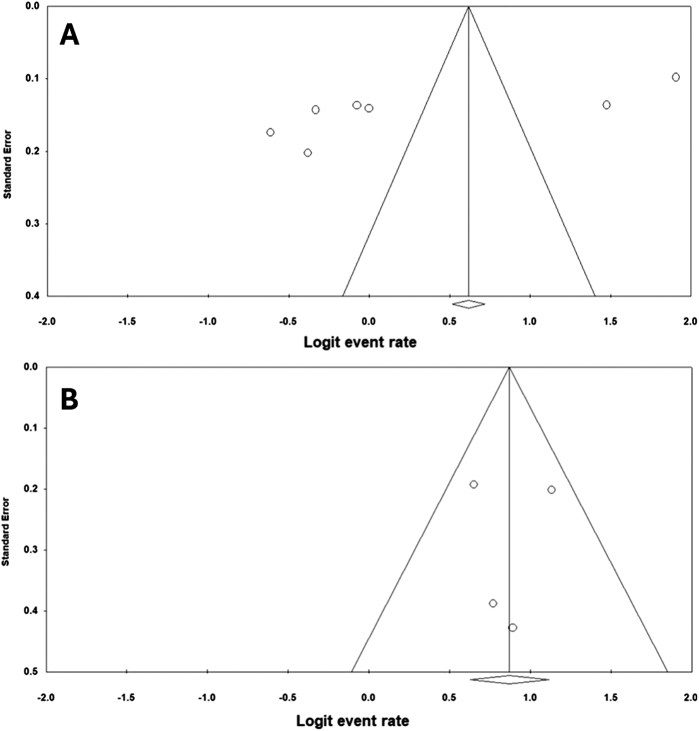
The funnel plots for (A) pooled prevalence studies; (B) odds ratio studies.

The potential presence of publication bias was examined using funnel plots for both the pooled prevalence and odds ratio. Figure 7(A) shows the funnel plot of the pooled prevalence studies, which is not visually symmetrical. Further testing was conducted using Egger’s test. The calculated *P*-value was 0.027, confirming the presence of publication bias. Trim and fill analysis was used, an adjusted pooled estimate of 72.23% (95% CI: 54.20%–85.16%) was calculated. Figure 7(B) demonstrates the funnel plot of the four odds ratio studies, which is visually symmetrical. After conduction of the Egger’s test, absence of publication bias was confirmed (*P*-value = 0.48).

### Subgroup analysis and meta-regression analysis

Table 3 shows the results of the subgroup analysis. Data collection by interview, the usage of Rome IV criteria for FD diagnosis, and the usage of higher PSQI cut-offs were linked with a higher pooled prevalence. However, this increase in prevalence was not statistically significant. The outcomes of the meta-regression analysis for mean age, percentage of females, sample size, publication year, and quality assessment score are shown in Table 4. Only the sample size showed a statistically significant association with prevalence estimates (regression coefficient = 0.003, *P* < 0.001), indicating that an increase in sample size is translated into an increase in the reported prevalence for PSQ.

**Table 3:** Subgroup analysis for different study categories

Subgroup analysis	Included groups	Number of papers	*I* ^2^ (%)	Pooled prevalence (%)	Upper limit (%)	Lower limit (%)	*P*-value	*Q* (*P*-value)
Method of data collection	Questionnaire	4	97.72	51.1	88.1	26.9	0.94	0.42 (0.516)
	Interview	3	99.98	64.9	88.1	38.8	0.38	
FD diagnosis	Rome III	5	98.7	53	77.4	27.1	0.83	0.32 (0.573)
	Rome IV	2	98.4	66.8	90.2	30.6	0.37	
PQI cut-off	>5/>5.5/≥6	4	97.45	53.1	75.2	29.8	0.80	0.17 (0.676)
	>8/≥8/>8.5	3	99.13	62.3	88.3	26.7	0.52

**Table 4:** Meta-regression analysis for different variables

Variable	Studies included	Regression coefficient	Standard error	*P*-value
Mean age	5	−0.043	0.061	0.486
Female %	7	0.018	0.025	0.464
Sample size	7	0.003	0.001	<0.001
Publication year	7	0.139	0.146	0.350
Quality assessment	7	−0.362	0.411	0.378

## Discussion

This work was based on a total of 2138 FD patients pooled from seven studies carried out in four different countries, demonstrating the pooled prevalence estimate of PSQ in FD patients to be 57.2%. Hence, poor sleep quality could be regarded as a common finding for FD patients, as one in two patients suffers from it. A previous meta-analysis reported a pooled prevalence of 19.0% for poor sleep quality based on 376 824 participants from the Chinese general population, which is less than the reported prevalence of PSQ in patients with FD^[28]^. The calculated odds ratio of 2.39 highlights that FD patients are more susceptible to PSQ especially when they are put into comparison with those who do not have FD.

PSQ is not only limited to patients with functional dyspepsia but has also been reported in other functional gastrointestinal disorders. One study reported a 46.9% prevalence of PSQ in Saudi irritable bowel syndrome (IBS) patients diagnosed using Rome IV^[29]^. Another study based on UK IBS patients reported that 66% of IBS patients recruited from a tertiary neuroethology clinic has PSQ^[30]^. Another study conducted on Japanese patients with gastroesophageal reflux disease (GERD) reported poor sleep in 52.2% of the patients^[31]^. The exact etiology of poor sleep in FGID patients is still not well understood; however, it could be due to the increased nocturnal arousal of the autonomic nervous system in FGID patients, resulting in poor sleep^[32]^. In addition, poor sleep was observed to be more severe with increased symptom severity and frequency in FGID, possibly by changing the pain thresholds, resulting in worsening of the patients’ condition, suggesting a bidirectional association between the two^[33,34]^.

Among the seven included studies, the highest prevalence was 87.1% as stated by Zhao *et al.* based on Chinese FD patients, conversely the lowest prevalence was 35.2% as stated by Yamawaki *et al.* based on Japanese FD patients. A previous observational study conducted on 26 851 individuals from the general population found that different sociodemographic factors affect the sleep and sleep quality of individuals including sex, residence, marital status and education, in addition to some habits like smoking and alcohol drinking^[35]^. Previous research has also found a close association between the occurrence of anxiety and depression with sleep quality^[36–38]^. The differences in distribution of the previously mentioned factors across different samples and populations could explain some of the variability in the reported prevalence. In addition, the severity of symptoms among FD patients could play a role. The 87.1% stated by Zhao *et al.* was calculated using a sample from class-three hospitals which are usually preserved for more severe and symptomatic patients. In FGID patients, sleep problems were more profound in patients reporting more severe symptoms or having pain that is interfering with their lives^[39]^. Additionally, 94.6% of these patients were reported to have coexisting psychological problems that could be associated with poor sleep^[40]^.

Subgroup analysis showed a comparable prevalence of PSQ in FD patients with respects to the method of data collection, with a slightly higher prevalence among studies using an interview instead of a self-administered questionnaire. The slight increase could be due to overreporting of the symptoms when an interview was conducted, possibly due to an interviewer bias or due to the clarification of symptoms to the patients. Subgroup analysis also showed a higher prevalence of poor sleep when using the most recent Rome criteria for the diagnosis of FD than when using the older Rome criteria. One of the modifications that were added to the Rome IVI criteria over the Rome III is the addition of the word “bothersome” to the four characteristic symptomologies of FD, which is defined as having symptoms that are severe enough to impact person’s daily activities indicating more symptoms severity as compared to Rome III^[41]^. This could explain the slight increase in poor sleep prevalence in Rome IV due to the increased threshold for symptom severity, with a resultant impairment of patients’ sleep. In addition, In Rome III, FD could be diagnosed without meeting a minimum frequency of symptom occurrence, which was refined in the Rome IV criteria, which requires a minimum frequency of dyspeptic symptoms before a diagnosis of FD is made. Apart from the differences in symptom severity, using the Rome IV criteria over the Rome III criteria decreases the overlap between different FD groups which includes post prandial distress syndrome (PDS) and epigastric pain syndrome (EPS) with an increased ability of classifying the patients accurately across these two groups^[42]^. Two of the included studies investigated the difference in prevalence of poor sleep across FD groups and no significant difference was found^[25,26]^.

### Limitations and directions for future research

With respect to our best knowledge, this work is the first to offer a pooled estimate of prevalence for PSQ in FD patients; however, several limitations are evident. First, despite the usage of the PSQI in all the included studies, studies used different cut-off values that could be associated with overestimation or underestimation of the true prevalence. Second, although it is recommended to assess for publication bias when the number of the studies is at least 10, our assessment of the publication bias highlighted the need for future research in this area to overcome the conflicting estimates. Third, despite the close association between psychological troubles, including depression, anxiety and non-specified psychiatric symptoms, and poor sleep quality, some of the included studies did not stratify or exclude patients with respect to these disorders, which limited our ability to conduct a subgroup analysis for the moderating effect of these variables on the occurrence of PSQ. Fourth, high heterogeneity was found across the seven studies assessing PSQ prevalence and the *I*^2^ values remained >90% even after the subgroup analysis. Lastly, as this analysis was based on cross-sectional studies in addition to the reported bidirectional association between PSQ and FGID, the exact cause–effect relationship cannot be concluded.

We recommend that future researchers use validated methods for disease identification, such as the PSQI and Rome criteria, in addition to using a filtering cut-off value of greater than 5 for the total PSQI, as it was originally identified as suitable for dividing the population into PSQ and good sleep quality groups. We also recommend the exclusion of patients with associated severe anxiety or depression and exclusion of subjects with an overlap between FD and other FGID to obtain more accurate estimations. In addition, we recommend grouping the patients according to their symptom severity and providing a separate estimate for each group, so that the burden of symptom severity can be assessed. Finally, we recommend using objective methods for sleep assessment, such as polysomnography, to eliminate the possibility of reporting and interviewer bias associated with subjective data collection methods.

## Conclusion

The problem of poor sleep quality is evident in FD patients. FD patients are at higher risks of poor sleep quality when compared to the healthy general population. Publication bias, high heterogeneity, in addition to differences in PSQI cut-offs, highlight the need for more rigorous studies to achieve an accurate disease estimate.

## Data Availability

Data sharing not applicable as no new data were generated or analyzed in the study.

## Research registration unique identifying number (UIN)

PROSPERO CRD42025644814.

## Significance statement

With respects to our best knowledge, this work is the first to offer a pooled estimate of prevalence for PSQ in FD patients. Our study found that half of the patients affected by functional dyspepsia (FD) experience poor sleep quality, with a pooled prevalence of 57.2%, making them more than twice as likely to have sleep disturbances compared to healthy individuals. Sleep disturbances are not only common but may exacerbate FD symptoms, creating a bidirectional relationship that worsens both conditions. This study underscores the need for standardized diagnostic criteria and refined methodologies in future research to better understand and manage the sleep-related challenges faced by FD patients.
